# Real-time guiding by deep learning during echocardiography to reduce left ventricular foreshortening and measurement variability

**DOI:** 10.1093/ehjimp/qyad012

**Published:** 2023-08-01

**Authors:** Sigbjorn Sabo, Hakon Neergaard Pettersen, Erik Smistad, David Pasdeloup, Stian Bergseng Stølen, Bjørnar Leangen Grenne, Lasse Lovstakken, Espen Holte, Havard Dalen

**Affiliations:** Department of Circulation and Medical Imaging, Norwegian University of Science and Technology, NTNU, Box 8905, 7491 Trondheim, Norway; Department of Circulation and Medical Imaging, Norwegian University of Science and Technology, NTNU, Box 8905, 7491 Trondheim, Norway; Department of Internal Medicine, Kristiansund Hospital, More and Romsdal Hospital Trust, Herman Døhlens vei 1, 6508 Kristiansund, Norway; Department of Circulation and Medical Imaging, Norwegian University of Science and Technology, NTNU, Box 8905, 7491 Trondheim, Norway; Sintef Digital, Box 4760 Torgarden, 7465 Trondheim, Norway; Department of Circulation and Medical Imaging, Norwegian University of Science and Technology, NTNU, Box 8905, 7491 Trondheim, Norway; Clinic of Cardiology, St Olavs University Hospital, Box 3250 Torgarden​, 7006 Trondheim, Norway; Department of Circulation and Medical Imaging, Norwegian University of Science and Technology, NTNU, Box 8905, 7491 Trondheim, Norway; Clinic of Cardiology, St Olavs University Hospital, Box 3250 Torgarden​, 7006 Trondheim, Norway; Department of Circulation and Medical Imaging, Norwegian University of Science and Technology, NTNU, Box 8905, 7491 Trondheim, Norway; Department of Circulation and Medical Imaging, Norwegian University of Science and Technology, NTNU, Box 8905, 7491 Trondheim, Norway; Clinic of Cardiology, St Olavs University Hospital, Box 3250 Torgarden​, 7006 Trondheim, Norway; Department of Circulation and Medical Imaging, Norwegian University of Science and Technology, NTNU, Box 8905, 7491 Trondheim, Norway; Clinic of Cardiology, St Olavs University Hospital, Box 3250 Torgarden​, 7006 Trondheim, Norway; Department of Internal Medicine, Levanger Hospital, Nord-Trøndelag Hospital Trust, Kirkegata 2, 7601 Levanger, Norway

**Keywords:** echocardiography, apical foreshortening, artificial intelligence, ejection fraction, global longitudinal strain

## Abstract

**Aims:**

Apical foreshortening leads to an underestimation of left ventricular (LV) volumes and an overestimation of LV ejection fraction and global longitudinal strain. Real-time guiding using deep learning (DL) during echocardiography to reduce foreshortening could improve standardization and reduce variability. We aimed to study the effect of real-time DL guiding during echocardiography on measures of LV foreshortening and inter-observer variability.

**Methods and results:**

Patients (*n* = 88) in sinus rhythm referred for echocardiography without indication for contrast were included. All participants underwent three echocardiograms. The first two examinations were performed by sonographers, and the third by cardiologists. In Period 1, the sonographers were instructed to provide high-quality echocardiograms. In Period 2, the DL guiding was used by the second sonographer. One blinded expert measured LV length in all recordings. Tri-plane recordings by cardiologists were used as reference. Apical foreshortening was calculated at the end-diastole. Both sonographer groups significantly foreshortened the LV in Period 1 (mean foreshortening: Sonographer 1: 4 mm; Sonographer 2: 3 mm, both *P* < 0.001 vs. reference) and reduced foreshortening in Period 2 (2 and 0 mm, respectively. Period 1 vs. Period 2, *P* < 0.05). Sonographers using DL guiding did not foreshorten more than cardiologists (*P* ≥ 0.409). Real-time guiding did not improve intra-class correlation (ICC) [LV end-diastolic volume ICC, (95% confidence interval): DL guiding 0.87 (0.77–0.93) vs. no guiding 0.92 (0.88–0.95)].

**Conclusion:**

Real-time guiding reduced foreshortening among experienced operators and has the potential to improve image standardization. Even though the effect on inter-operator variability was minimal among experienced users, real-time guiding may improve test–retest variability among less experienced users.

**Clinical trial registration:**

ClinicalTrials.gov, Identifier: NCT04580095.

## Graphical Abstract

**Graphical Abstract:**
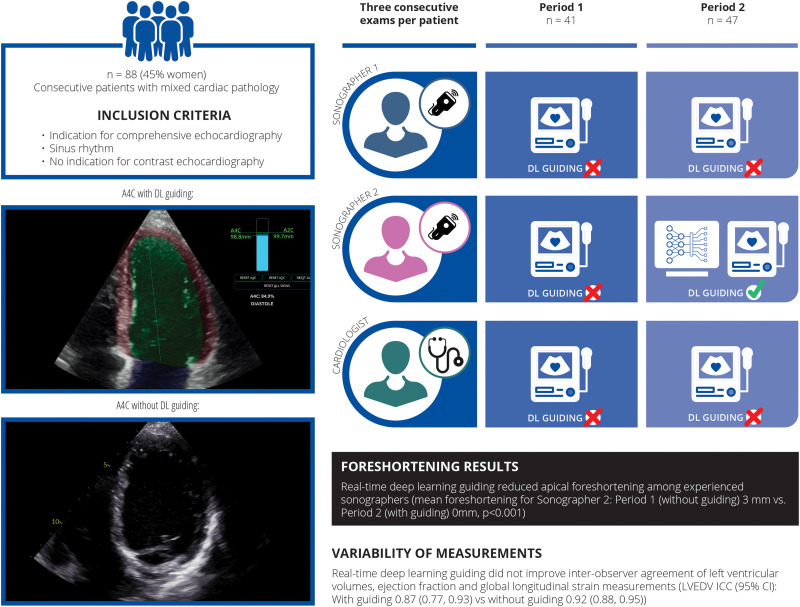
Visual summary of participant recruitment, study design, and central results. A4C, apical four-chamber; DL, deep learning; ICC, intra-class correlation coefficient; LVEDV, left ventricular end-diastolic volume.

## Introduction

Echocardiography is the most commonly used imaging modality in cardiology and provides essential information about cardiac function and morphology. Despite this, subjective preferences and suboptimal image standardization make echocardiography operator-dependent, with considerable variability even in the hands of experienced operators.^1–3^ Thus, the European and American cardiology societies highlight the importance of standardized image acquisition and adequate image quality for achieving the best possible results in echocardiography.^4,5^

Foreshortening of the left ventricle (LV) is a common problem in echocardiography.^6^ Two-dimensional echocardiographic measurements depend on geometrical assumptions of the three-dimensional ventricle, making geometric distortion a frequent pitfall. Some effects of foreshortening are overestimation of systolic LV global longitudinal strain (GLS) and ejection fraction (EF), while LV length and volumes are underestimated.^6^ The relationship between foreshortening and the error introduced in EF calculations has been shown to be U-shaped, highlighting the need to minimize foreshortening during echocardiographic scanning.^7^

Deep learning (DL) allows for real-time analysis and quantification of LV function and geometry.^7,8^ Most publications investigating DL tools within echocardiography have targeted *post hoc* image analyses and automatic measurements.^9–11^ To improve the quality and standardization of images, real-time guiding during acquisition may be beneficial.^12^ This is of the highest clinical importance for every echocardiographic laboratory. Real-time guiding of operators is a novel field of research, with some publications investigating support for novice operators.^13,14^ However, no publication has investigated its ability to optimize recordings with experienced operators. Thus, in this study, we sought to investigate the effect of real-time feedback of LV length and foreshortening metrics during image acquisition by experienced sonographers using a robust DL-based tool to reduce apical foreshortening. Furthermore, we aimed to evaluate the effect of the real-time feedback on LV length and foreshortening on test–retest variability of LV end-diastolic volume (LVEDV), EF, and GLS.

## Methods

### Study population

Patients with mixed cardiac pathology were prospectively recruited from the echocardiography laboratory at St Olavs University Hospital. The inclusion was restricted to specific days when dedicated equipment and personnel were available at the laboratory. On average, five patients were included each day. The inclusion criteria were (i) indication for comprehensive echocardiography and (ii) ability to provide written informed consent. Patients with non-sinus rhythm or indication for contrast echocardiography were excluded. This study was approved by the regional ethical committee (REC Central Norway 7160) and performed according to the Helsinki declaration.

### Study design

All participants underwent three consecutive echocardiograms without leaving the examination bench (*Graphical Abstract*). The participants stayed supine between examinations, and the time delay between examinations was minimized. The first and second examinations were performed by two (of three) experienced sonographers, and the third examination by one (of four) cardiologist experts. The sonographers were randomly allocated to perform the first examination (Sonographer 1) or the second examination (Sonographer 2). Thus, the sonographer using the guiding tool in Period 2 was not the same individual in all inclusions but was randomly drawn from the three participating sonographers at each patient inclusion to account for differences in operator experience. Similarly, the selection of a cardiologist for the reference examination was random. Before the study, sonographers and cardiologists had acquired a minimum of 2000 and 10 000 echocardiograms, respectively.

**Figure 1:**
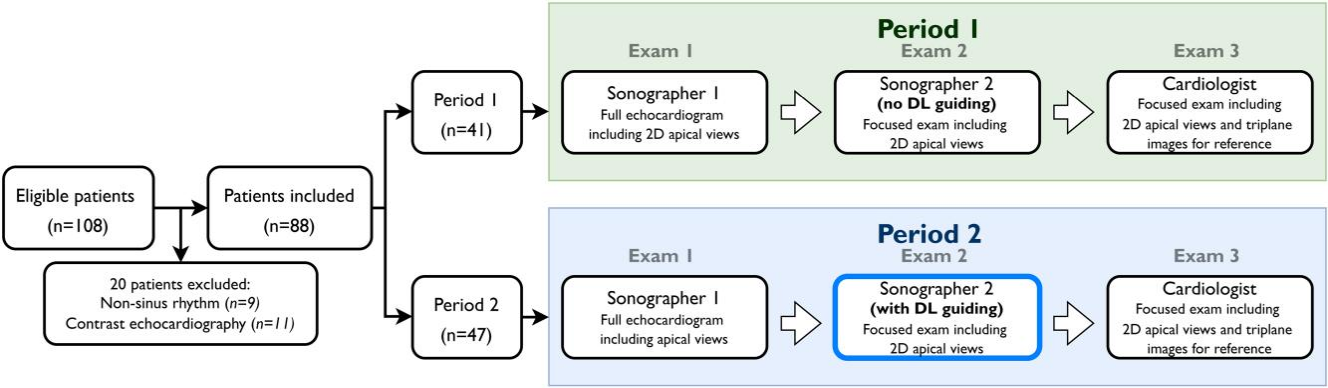
Flow chart of the study population. Participant inclusion process is illustrated in the left part of the figure. The two panels indicate the two separate study periods (Period 1 and Period 2). The second examination (thickened frame) in Period 2 illustrates the sonographers using the real-time deep-learning algorithm. DL, deep learning; 2D, two-dimensional.

The data collection process was divided into two periods, each lasting 2 months between September 2020 and June 2021 (*Figure 1*). In the first period (Period 1), the sonographers were instructed to provide echocardiograms optimized for between-operator comparison of measurements of LV size and function. However, they were not informed about the aim of the study or the content of the second data collection period. After Period 1, the sonographers were introduced to the real-time DL tool and trained in its use on 10 patients each. In the second period (Period 2), the sonographer who performed the second examination used the real-time DL tool during scanning, while the first sonographer acquired the echocardiograms as in Period 1 (except for recently being trained in the use of the DL tool and being aware of the study aims).

### DL foreshortening detection algorithm

**Figure 2:**
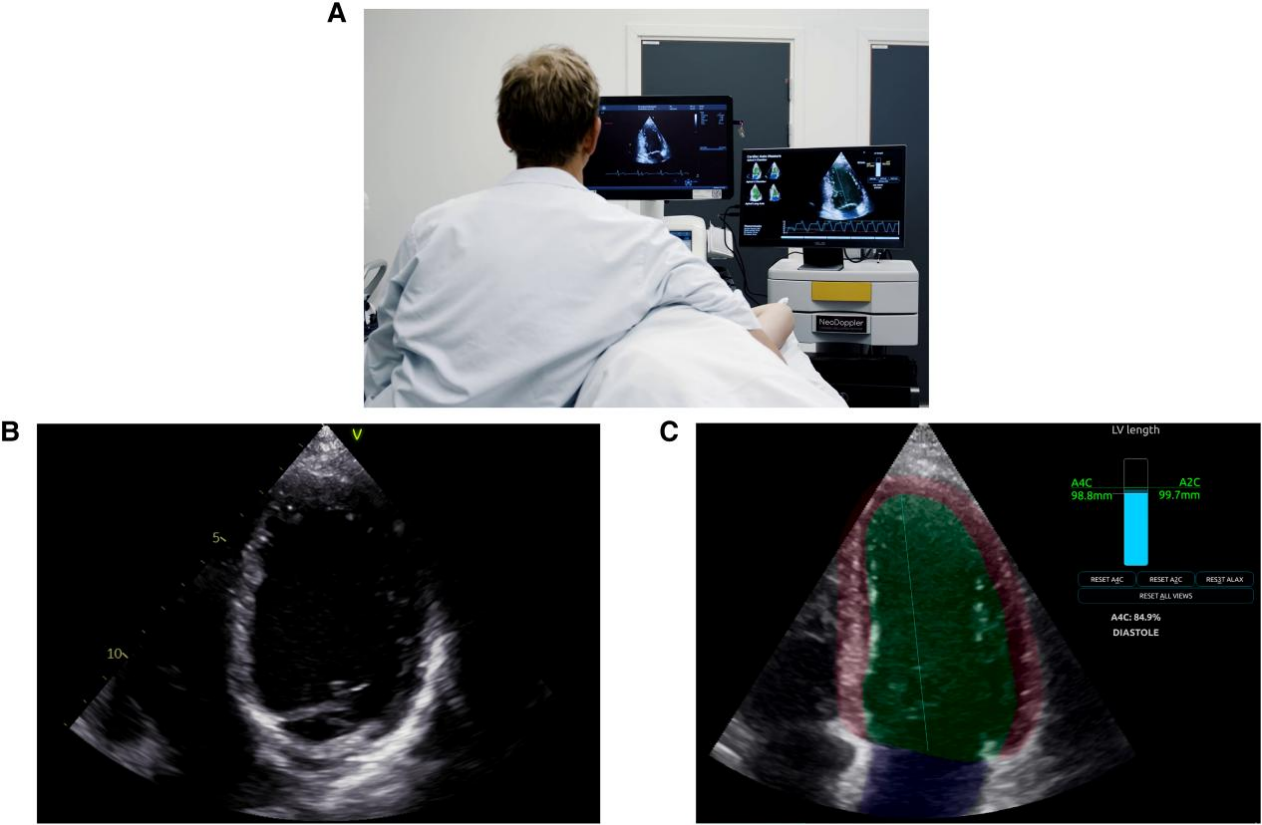
The real-time deep-learning feedback of view-specific left ventricular length and apical foreshortening. (*A*) The real-time DL-guiding tool (right screen) adjacent to the ultrasound machine (left screen). The tool is also visualized in Supplementary data online, *Video S1*. (*B* and *C*) Screenshots of the DL real-time foreshortening detection from four-chamber (A4C) recordings of the same patient. (*B*) Foreshortened A4C. (*C*) A4C recording using the DL-guiding tool without foreshortening (no significant difference between the A4C LV length and corresponding A2C LV length in the LV length indicator to the right in the image).

The DL algorithm operated in real-time during scanning (*Figure 2* and see Supplementary data online, *Video S1*). Details regarding the training, structure, and function of the DL algorithm have been published previously.^7^ In short, separate DL networks performed cardiac view classification, timing of cardiac events, segmentation of the LV, and landmark extraction. Apical foreshortening was quantified using landmark extraction, which derived the LV length from apical and basal landmarks. The maximal length across cycles and views was saved and presented visually. If the LV length difference between views exceeded 3 mm, the LV line colour changed from green to yellow. In addition, longitudinal movement of the apex was calculated and presented by a pop-up warning if a threshold of 8 mm was exceeded (*Figure 2C*). While scanning, the operator was allowed to revise the recordings in case of LV foreshortening. If additional recordings were obtained due to foreshortening, the most appropriate was used in the analyses. During data collection, the real-time foreshortening algorithm was set up on a separate computer with the monitor placed adjacent to the ultrasound machine allowing for real-time streaming from the ultrasound machine (*Figure 2A*).

### Echocardiographic image acquisition

Examinations were performed using a Vivid E95 scanner (version 202; GE Vingmed Ultrasound, Horten, Norway) with a phased array transducer (4Vc). For each recording, three consecutive cardiac cycles were obtained. The first examination was a comprehensive echocardiogram according to ASE/EACVI guidelines.^4^ The second and third examinations were focused and included 2D greyscale apical two-, four-, and three-chamber recordings for analyses of LV size and function. Furthermore, the cardiologists’ examination included tri-plane recordings for reference measurements of LV length. All study-specific recordings were blinded and de-identified before storing.

### Analyses of LV size and function

All measurements were performed using EchoPAC SWO (Versions 203 and 204; GEVingmed Ultrasound, Horten, Norway). The LV endocardial borders were traced at the end-diastole and end-systole in apical four- (A4C) and two-chamber (A2C) recordings. LV volumes and EF were calculated using the method of disc summation.^4^ For calculations of biplane volume and EF in EchoPAC SWO, the maximal LV length from A4C or A2C was used. Each operator analysed their own recordings blinded to all other operators.

GLS was measured using the Automated Functional Imaging package from the three standard apical views in an 18-segment model.^4^ To adjust for less myocardium in the apical region, GLS and regional longitudinal strain (apical, mid-ventricular, and basal levels) were calculated in a 16-segment model: The basal and mid-ventricular levels included six segments each, while apical strain was based on four segments averaging the values from the anteroseptal and inferoseptal walls and the inferolateral and anterolateral walls.

**Figure 3:**
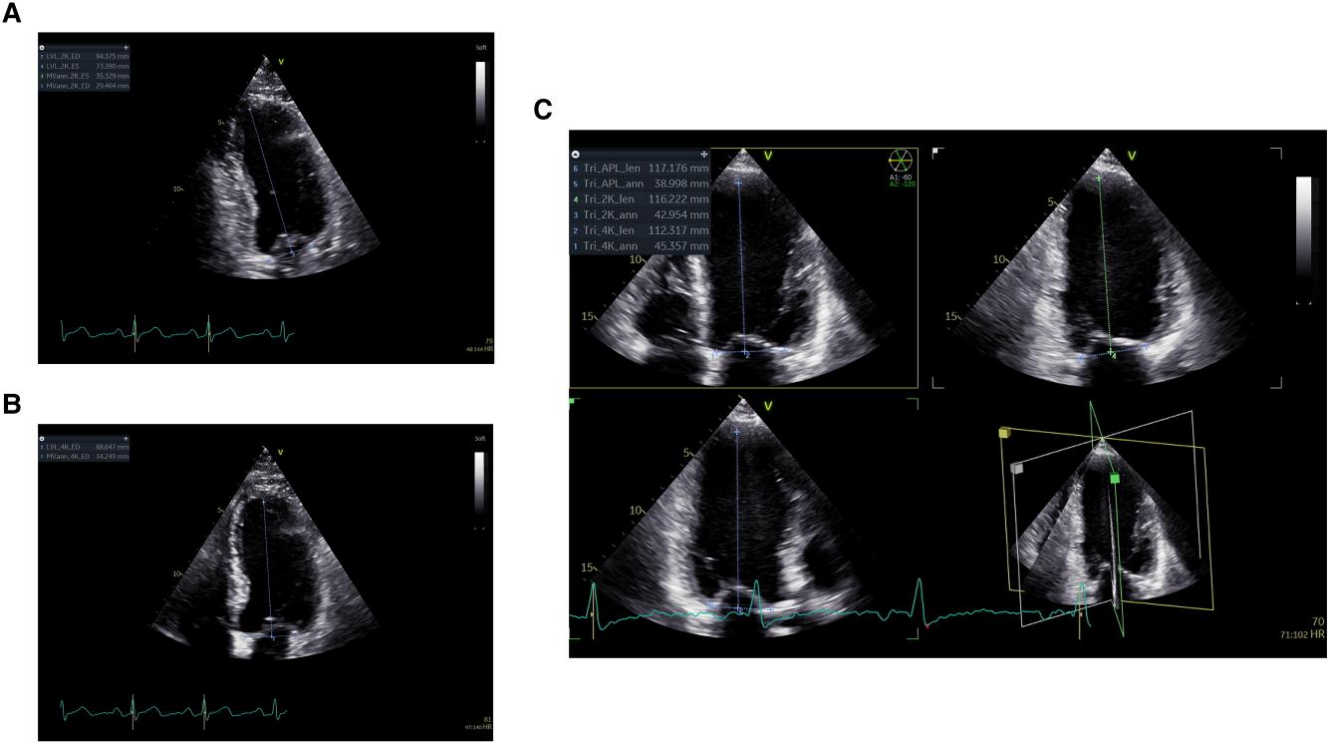
LV length measurement method. Screenshots of calipers used for measuring LV length at the end-diastole. First, a caliper is placed between the two mitral annular points. Secondly, the LV length is estimated by placing another caliper from the subendocardial apical point to the centrum of the first mitral annular caliper. (*A*) Apical two-chamber recording. (*B*) Apical four-chamber recording. (*C*) Cardiologist’s tri-plane recording used for reference LV length. The mean LV length of the three planes was used as reference. The minor differences between views are partly explained by mispositioning of the apical point in four-chamber view, while the mitral annular curvature may also explain some variability between views.

LV length was measured in 2D apical views recorded by all three operators and in the cardiologists’ tri-plane recordings by an expert reader (H.D.; *Figure 3*). Two-dimensional images were analysed blinded to patients’ and operators’ characteristics separately from the tri-plane recordings. The LV length was measured at the end-diastole, applying a straight line from the subendocardial apical point to the middle part of a straight line between the two mitral annular points. The average LV length from the cardiologist tri-plane recording was defined as the participants’ reference LV length. Foreshortening was calculated as the reference LV length from tri-plane recordings minus the view-specific LV length from 2D recordings. Foreshortening is reported per view and as averaged values. Operator-specific echocardiograms were dichotomized to foreshortened or not using an arbitrary cut-off of ≥5 mm difference between reference and operator-specific LV length. The LV length was also derived from the LVEDV measurement made by each operator to investigate the association of LV traces’ length with the reference LV length. The reproducibility of the reference LV length measurements was evaluated by reanalysing all echocardiograms from Period 1 by another expert reader (E.H.) utilizing the methodology described above.

### Statistics

Normally distributed continuous variables are presented as means and standard deviations (SDs). Proportions are presented as numbers (%). In this study, sonographer acquisitions were grouped by guiding (*n* = 47) vs. no guiding (*n* = 129) and compared against the cardiologist’s reference. The intra-class correlation coefficient (ICC), defined as the proportion of the between-individual variance to the total variation, was used to assess the inter-observer agreement of systolic measurements.^15,16^ The ICC was estimated using a linear mixed-effects model,^16^ treating individual study participants as random effects and operator groups as fixed effects. A bootstrap (1000 samples) was used to estimate a 95% confidence interval (CI) for the ICC. The normality of error terms was inspected visually with Q-Q plots, and logarithmic transformation was applied where necessary. The differences in measurements between Periods 1 and 2 were analysed using independent sample *t*-tests. For differences between operator groups and within periods, a linear mixed model was used in the same manner as above. Fisher’s exact test was used for ascertaining the differences in the proportions of the foreshortened images. A linear mixed model was used to assess the relationship between apical foreshortening and difference in LV length measured by the expert reader and the LV length derived from the LVEDV measurement. A *P*-value of <0.05 was considered statistically significant. All statistical analyses were performed by using R 4.2.2 (RStudio, packages lme4 and lmerTest). Sample power was prospectively estimated using SamplePower 3.0 (SPSS Inc., Chicago, IL, USA). We estimated 90% power (*α* = 0.05) to detect a between-group difference of 1 mm (SD 3 mm) foreshortening using the DL tool by the sonographer groups with a between-measurement correlation of 0.8.

## Results

### Study population

Of 108 recruited participants, those not in sinus rhythm (*n* = 9) and needing contrast echocardiography (*n* = 11) were excluded. Thus, 88 patients (45% women) were included. A flow chart of the inclusion process is shown in *Figure 1*. Baseline characteristics of the study population and the main systolic measurements are presented in *Table 1*. In short, mean (SD) age was 63 (16) years, 42 (48%) had heart failure or previous myocardial infarction, while 17 (19%) had moderate or severe valvular disease. Only 3 (3%) had chronic obstructive pulmonary disease.

**Table 1:** Basic characteristics of the study population according to study periods

	Period 1	Period 2
Included study participants, *n*	41	47
Age	62 (18)	63 (15)
Women, *n* (%)	19 (45)	21 (46)
Clinical characteristics		
Heart failure, *n* (%)	8 (20)	15 (32)
Acute myocardial infarction, *n* (%)	10 (2)	8 (17)
Moderate or severe valvular disease, *n* (%)	7 (17)	10 (21)
Chronic obstructive pulmonary disease, *n* (%)	2 (5)	1 (2)
Body mass index, kg/m^2^	26 (4)	27 (6)
Heart rate, beats/min	66 (13)	69 (11)
Systolic blood pressure, mmHg	143 (25)	140 (21)
Echocardiographic characteristics		
LV ejection fraction, %	54 (13)	53 (12)
LV end-diastolic volume, biplane, mL	124 (53)	127 (56)
LV global longitudinal strain, %	−17 (5)	−16 (5)

Values are provided as mean (SD) or number (proportion).

LV, left ventricular.

Systolic LV measurements according to operators and study periods are summarized in *Table 2*. In short, sonographers measured significantly lower biplane end-diastolic volumes compared with cardiologists in both study periods (*P* ≤ 0.014). Furthermore, both sonographer groups measured significantly lower absolute GLS than cardiologists in Period 2 (*P* < 0.001).

**Table 2:** Left ventricular volume and systolic measurements according to operators and study periods

	Period 1	Period 2
Biplane LV end-diastolic volume, mL		
Cardiologist	123.8 (52.5)	127.4 (56.1)
Sonographer 1	118.4 (54.9)^a^	111.5 (54.2)^a^
Sonographer 2	117.2 (53.0)^a^	109.3 (52.9)^a^
Biplane LV ejection fraction, %		
Cardiologist	53.7 (13.1)	52.5 (11.8)
Sonographer 1	55.7 (12.7)	53.2 (14.0)
Sonographer 2	55.1 (13.3)	54.9 (12.6)
LV global longitudinal strain, %		
Cardiologist	−17.0 (4.6)	−16.0 (4.7)
Sonographer 1	−16.1 (4.7)	−15.5 (4.9)^a^
Sonographer 2	−16.0 (6.0)	−15.7 (4.6)^a^

Values are presented as mean (SD).

LV, left ventricular.

a Measurement by sonographer significantly different from cardiologist measurement.

### Real-time intervention to reduce LV foreshortening

**Figure 4:**
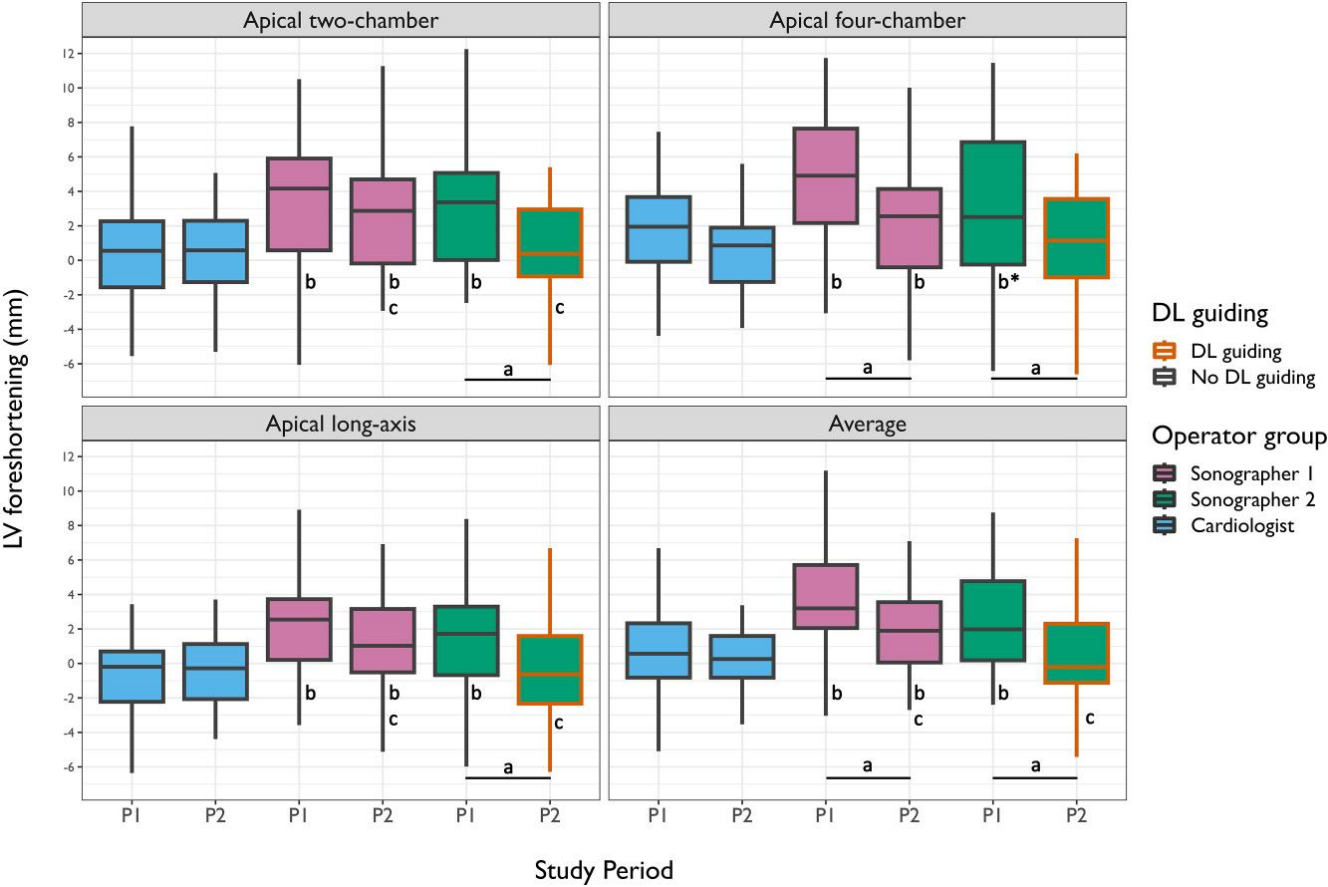
Box plots of apical foreshortening for each view and operator. Box plots for LV foreshortening grouped by study period, operator group, and use of DL guiding. Box plot centre line is the median with whisker lengths extending 1.5 times the interquartile range above Q3 and below Q1. a = *P* < 0.05 between study periods within the same operator group; b = *P* < 0.05 vs. cardiologist within same study period (except b*; *P* = 0.075), c = *P* < 0.05 vs. other sonographer group within same study period (cardiologists excluded). LV, left ventricular; P1, Period 1; P2. Period 2; DL, deep learning.

Apical foreshortening of the LV recordings is shown in *Table 3* and graphically in *Figure 4*, according to operators and study periods. Compared with the reference cardiologist, acquisitions by Sonographers 1 and 2 were significantly foreshortened in Period 1 (mean foreshortening 4 and 3 mm, both *P* < 0.001). In Period 1 (no DL tool), the foreshortening was most pronounced (3–5 mm) in A4C and A2C views (difference vs. reference; A4C for Sonographer 2, *P* = 0.075; others *P* < 0.001) compared with the long-axis view (1–2 mm difference vs. reference; both *P* < 0.01). LV foreshortening was reduced in Period 2 compared with Period 1 for sonographers with and without DL guiding. Mean changes were 1 mm for Sonographer 1 without the DL tool and 2 mm for Sonographer 2 with the DL tool (both *P* ≤ 0.02). Compared with the expert cardiologists, the LV recordings in Period 2 by Sonographer 1 (no DL tool) were still significantly foreshortened (all views, *P* < 0.01). In contrast, the recordings by Sonographer 2 (with the DL tool) were not foreshortened (all views, *P* > 0.41). Furthermore, both sonographer groups had numerically higher proportions of foreshortened examinations than cardiologists in Period 1 [Sonographer 1, 12 (29%), *P* = 0.048; Sonographer 2, 10 (24%), *P* = 0.140; cardiologist, 4 (10%)]. In Period 2, Sonographer 1 foreshortened more examinations compared with the cardiologists [7 (23%) vs. 0 (0%), *P* = 0.012], while Sonographer 2 did not [1 (4%) vs. 0 (0%), *P* = 1]. The proportion of foreshortened recordings was significantly reduced between periods only for Sonographer 2 (*P* = 0.002).

**Table 3:** Left ventricular foreshortening according to operators, views, and study periods

	Period 1		Period 2		Period 1 vs. Period 2
	mm (SD)	*P*-value [vs. reference]	mm (SD)	*P*-value [vs. reference]	*P*-value
Foreshortening, averaged					
Sonographer 1	3.5 (3.1)	<0.001	2.1 (2.6)	<0.001	0.023
Sonographer 2	2.6 (3.0)	<0.001	0.5 (2.5)	0.409	<0.001
Cardiologist (reference)	0.6 (2.9)		0.2 (1.7)		0.422
Foreshortening, A2C					
Sonographer 1	3.2 (4.6)	<0.001	2.8 (3.4)	<0.001	0.645
Sonographer 2	3.4 (4.0)	<0.001	0.6 (3.2)	0.536	<0.001
Cardiologist (reference)	0.4 (3.6)		0.3 (2.9)		0.908
Foreshortening, A4C					
Sonographer 1	4.7 (4.3)	<0.001	2.0 (3.4)	0.009	0.002
Sonographer 2	3.1 (4.6)	0.075	1.0 (3.4)	0.452	0.016
Cardiologist (reference)	1.8 (4.0)		0.6 (2.4)		0.077
Foreshortening, APLAX					
Sonographer 1	2.4 (3.8)	<0.001	1.3 (3.9)	0.003	0.176
Sonographer 2	1.3 (3.3)	0.008	−0.2 (3.0)	0.751	0.029
Cardiologist (reference)	−0.5 (2.8)		−0.4 (2.2)		0.842

Values are presented as mm (SD). *P*-values for Period 1 vs. Period 2 were calculated using independent sample *t*-tests. *P*-values for within period differences between operators were calculated using mixed linear models.

A2C, apical two-chamber view; A4C, apical four-chamber view; APLAX, apical long-axis view.

### LV foreshortening and variability of measurements

The inter-observer agreement ICCs (for comparison with reference) showed non-significant differences between sonographers with and without the DL algorithm (*Table 4*). The 95% CIs overlapped for LV volume in biplane and single-view recordings and for LV EF and LV GLS. ICCs were in the range of 0.77–0.94. The ICCs were lowest for LV EF and highest for LV GLS. The difference in LV length between A4C and A2C was significantly associated with lower LVEDV in the foreshortened view (4.2 mL difference in LVEDV per 5 mm difference in length, *P* = 0.033).

**Table 4:** Intra-class correlation coefficients for agreement against cardiologist measurement between sonographers with and without guiding

	ICC (95% CI)	
	With guiding	Without guiding
LV ejection fraction, biplane	0.77 (0.63–0.87)	0.83 (0.74–0.89)
LV global longitudinal strain	0.94 (0.90–0.97)	0.91 (0.87–0.94)
LV end-diastolic volume, biplane	0.87 (0.77–0.93)	0.92 (0.88–0.95)
LV end-diastolic volume, A4C	0.83 (0.73–0.91)	0.90 (0.85–0.94)
LV end-diastolic volume, A2C	0.83 (0.72–0.90)	0.87 (0.80–0.92)

ICC point estimates were calculated using mixed linear models. A bootstrap was used to estimate 95% confidence intervals (CIs).

A2C, apical two-chamber view; A4C, apical four-chamber view; ICC, intra-class correlation coefficient; LV, left ventricular.

**Figure 5:**
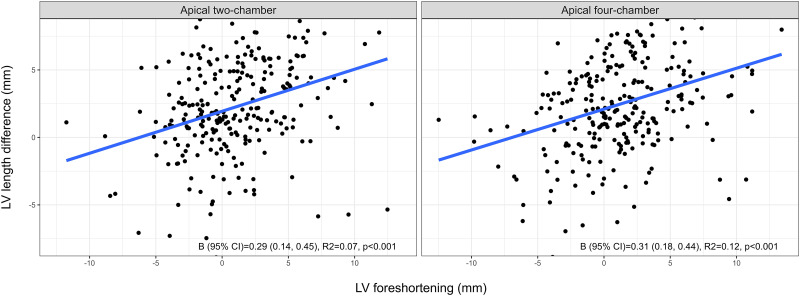
Scatter plot with a linear mixed model regression line. LV length difference in relation to LV foreshortening in sonographer examinations. LV foreshortening (*x*-axis) was calculated as the reference LV length obtained from the same 2D recording minus the reference LV length from the cardiologist’s tri-plane recording. LV length difference (*y*-axis) was calculated as LV length extracted from the view-specific end-diastolic volume measurement minus the reference length obtained from the same 2D recording. LV, left ventricular.

Additionally, there was a positive association between LV foreshortening and the observer’s manual length measurement during assessment of LVEDV (*Figure 5*), indicating that the sonographers compensated for foreshortened LV recordings during analyses. The sonographers traced the LV 0.3 mm longer (compared with reference) per 1 mm foreshortening of the apical two- and four-chamber recordings (*P* < 0.001). Moreover, sonographers accepted more GLS segments than cardiologists, with a difference of mean (SD) 1.3 (0.2) segments (*P* < 0.001).

The inter-observer agreement for the reference LV length method was excellent with a mean (SD) absolute difference between the two expert readers of 1.4 (1.7) mm and ICC (95% CI) of 0.98 (0.95–0.99).

## Discussion

This study investigated the effect of real-time DL guiding during echocardiographic scanning to reduce apical foreshortening within experienced operators. The main finding of this study was the significant reduction of apical foreshortening among experienced sonographers who used the real-time DL tool, suggesting that guiding during scanning can standardize echocardiographic acquisition and improve image quality even in experienced sonographers. However, compared with cardiologists’ reference measurements, the inter-observer agreement of the sonographers’ analyses for LV volume, EF, and GLS was not improved.

### Population

The study population consisted of prospectively recruited patients with mixed cardiac pathology, only excluding patients in non-sinus rhythm or with indications for contrast due to impaired image quality. The inclusion was limited to specific days due to study logistics. Even though the population is limited, we suggest that our findings may be generalized to similar populations commonly found in echocardiographic units. The sonographers were experienced with more than 5 years of echocardiographic experience and performance of >2000 comprehensive echocardiograms each. Similarly, the cardiologist experts performing the reference examinations were highly experienced and well-informed about the study aims. The results show that real-time guiding can improve image acquisition even among highly experienced operators, indicating a potential clinical benefit of implementing DL tools for real-time guidance. The effect may be more pronounced in less experienced operators, but this must be evaluated in future studies.

### Real-time intervention to reduce LV foreshortening

Using the real-time DL guiding tool, sonographers significantly reduced apical foreshortening of LV acquisitions compared with those not using the real-time DL tool. Even though the foreshortening was only a few millimetres, the foreshortening was minimalized by use of the real-time DL guiding tool. To the best of our knowledge, no other study has investigated the effect of real-time guiding of experienced operators by a novel DL tool during echocardiographic scanning. However, two studies have evaluated the use of tools for guiding inexperienced users during cardiac ultrasound. Narang *et al.*^13^ and Schneider *et al.*^14^ used machine-learning-based image quality scores to aid the operator during echocardiographic acquisition. Both studies showed that real-time guiding of operators without echocardiographic experience resulted in images of diagnostic quality in most examinations. Furthermore, the effect of guiding on inter-observer agreement and measures of LV foreshortening in experienced operators remains uninvestigated until now.

Our results show that real-time feedback during echocardiographic scanning can reduce errors in echocardiographic recordings, even among experienced operators. Furthermore, the sonographers not using the DL tool also reduced foreshortening in Period 2. Thus, the emphasis on apical foreshortening and training in use of the DL tool between the two inclusion periods improved the standardization of the acquisitions. This highlights some of the difficulties researchers face when designing studies to investigate the effects of real-time guiding, as the control group often improves too. Importantly, sonographers without DL guiding in Period 2 still foreshortened the images more than expert cardiologists and sonographers using the DL tool, indicating the importance of continuous training and quality control in echocardiographic laboratories, as well as the impact of real-time guiding.

### Variability of LV measurements

No statistically significant effect of DL guiding on inter-observer agreement of systolic measures was found in our study. The analyses of the sonographers and the reference cardiologists both influence this measure, and avoiding apical foreshortening may have diverted operators from other parameters of image standardization, like endocardial border detection and segmental visualization of the myocardium. We believe reading effects introduced during analyses affected the variability of measurements more than the 2–5 mm of foreshortening in this study. The reading effect influences the variability of both the sonographers’ measurements and the cardiologist experts’ reference measurements and relates to differences in endocardial border tracing and manual positioning of apical and annular landmarks. It has been previously shown that the reading effect is more important than the acquisition effect for LV EF, while the acquisition seems more important for GLS analyses.^17^ Still, for the presented results by experienced operators, the reading effect seems to be more important than the foreshortening for measurements of both LV EF and GLS.

In this study, we found a significant association of apical foreshortening with the difference between LV length measured in the operators’ endocardial traces and by the reference. This may indicate that in foreshortened LV recordings, the operators compensate by positioning the apical point of the trace more into the myocardium, thereby increasing the LV length. The LVEDV and LV EF estimation by the biplane Simpson method in EchoPAC SWO also compensates for foreshortening by using the maximal LV length from the A4C or A2C view in the calculation. It was a significant association between the difference in foreshortening and the LVEDV estimates between the two views. Thus, the EchoPAC SWO measurement method may have modulated the effect of foreshortening on the variability of biplane volume measurements. Despite this, single-view analyses without LV length correction provided no substantial change and showed a non-significant effect of real-time guiding on measurement agreement.

Sonographers accepted significantly more cardiac segments than cardiologists in the GLS analyses. It is well known that different operators may initialize the region of interest for strain analyses differently. Furthermore, GLS reproducibility varies between studies, operators, and vendors.^17–19^ Thus, the present finding of no significant change concerning variability does not limit the need to optimize the recordings. In the future, automatic analyses of central LV function measurements may reduce the variability related to the reader effect.^11^ Automatic measurements by DL are without intra-observer variability.^11,20^ Together with more standardized recordings, this may improve the sensitivity to detect changes in cardiac morphology and function. Studies investigating the effect of real-time guiding and automatic measurements of central indices are therefore warranted.

### Strengths and limitations

The main strengths of the study are (i) the study design, with blinding of the two sonographer groups to the study aim until exposition for the real-time DL tool before the second study period, (ii) the blinding of the operators to details from the other operators’ recordings and analyses and blinded reference measurements of LV length, and (iii) the interface for implementing the DL tool into real-life echocardiographic scanning. The use of experienced operators has probably led to an underestimation of the effect on foreshortening and measurement variability compared to less experienced users. However, most sonographers in echocardiographic laboratories worldwide are experienced, and we believe that optimizing recordings is equally important in these scenarios. Another limitation is the modest sample size, which may have reduced the ability to detect subtle changes in inter-observer variability. Dichotomization of foreshortening using a predefined cut-off of ≥5 mm is a limitation and should be interpreted with care. This cut-off was used as an arbitrary limit indicating clinically significant foreshortening. Importantly, any foreshortening of the LV will pose challenges to correct measurements of size and function. As tri-plane views were recorded by using only the cardiologists’ reference examination, we do not know whether a more active use of tri-plane recordings would have reduced LV foreshortening.

## Conclusion

Real-time guiding by DL with feedback of LV length during echocardiographic scanning by experienced sonographers improved the standardization of recordings by reducing apical foreshortening. We found no significant effect of real-time guiding on the inter-observer agreement of LV measurements. However, more extensive studies with less experienced operators are needed to fully evaluate the effect of real-time guiding on the variability of echocardiographic measurements. Whether the combination of real-time guiding and automatic measurements will further reduce variability in echocardiography should be evaluated in future studies.

## Supplementary Material

qyad012_Supplementary_Data

## Data Availability

The data set can be made available from the corresponding author upon reasonable request.
